# The RNA M5C methyltransferase NSUN2 promotes progression of hepatocellular carcinoma by enhancing PKM2-mediated glycolysis

**DOI:** 10.1038/s41419-025-07414-5

**Published:** 2025-02-09

**Authors:** Qin Qi, Rui Zhong, Yan Huang, Yong Tang, Xiao-wen Zhang, Chang Liu, Chun-fang Gao, Lin Zhou, Jian Yu, Lu-yi Wu

**Affiliations:** 1 Yueyang Hospital of Integrated Traditional Chinese and Western Medicine, Shanghai University of Traditional Chinese Medicine, Shanghai, China; 2 International Joint Research Centre on Purinergic Signalling, Chengdu University of Traditional Chinese Medicine, Chengdu, China; 3 Department of Laboratory Medicine, Changzheng Hospital, Naval Medical University, Shanghai, China; 4 Department of Laboratory Medicine, Eastern Hepatobiliary Surgery Hospital, Naval Medical University, Shanghai, China; 5 The Third Department of Hepatic Surgery, Eastern Hepatobiliary Surgery Hospital, Naval Medical University, Shanghai, China

**Keywords:** Cancer genomics, Cancer metabolism

## Abstract

Hepatocellular carcinoma (HCC) is one of the most common cancers worldwide. The 5-methylcytosine (m5C) RNA methyltransferase NSUN2 is involved in cell proliferation and metastasis and is upregulated in a variety of cancers. However, the biological function and regulatory mechanism of NSUN2-mediated m5C modification have not been well studied in HCC. Our results showed that NSUN2 is upregulated and associated with poor prognosis in HCC patients after hepatectomy. NSUN2 overexpression significantly promoted HCC growth and metastasis, whereas NSUN2 knockdown had the opposite effect. m5C RNA immunoprecipitation sequencing (m5C-RIP-Seq) revealed that m5C hypermethylation correlates with mRNA overexpression and that NSUN2-mediated m5C hypermethylation promotes metabolism in HCC patients. Mechanistically, our data revealed that PKM2, a terminal enzyme in the glycolytic pathway, is a downstream target of NSUN2-mediated m5C modification. Specifically, NSUN2 could stabilize PKM2 mRNA by increasing the m5C level of the m5C site C773 in the 3′-UTR of PKM2 mRNA. In addition, rescue assays revealed that NSUN2 promotes HCC glycolysis and progression by upregulating PKM2. In conclusion, this study revealed that NSUN2-mediated m5C modification promotes glycolysis and the progression of hepatocellular carcinoma by stabilizing PKM2 mRNA, and provides a potential prognostic factor and therapeutic target for HCC patients.

## Introduction

Hepatocellular carcinoma (HCC) is the sixth most common cancer worldwide and the third leading cause of cancer death worldwide [1]. Surgical treatment, including hepatectomy and liver transplantation, is effective and can increase the chance of survival in HCC patients. However, owing to metastasis and recurrence after hepatectomy, the 5-year survival rate of HCC patients is still low. By studying the mechanism of the occurrence and development of HCC, it is possible to discover new biomarkers and find effective therapeutic targets for HCC patients. Thus, further exploration of the molecular mechanism of HCC progression is necessary.

To date, 172 types of RNA modifications have been identified [2]. Among them, RNA N6-methyladenosine (m6A) is the most pervasive, and many studies have reported its important role in HCC [3–6]. 5-Methylcytosine (m5C) is another abundant RNA modification and is widespread in ribosomal RNAs (rRNAs), transfer RNAs (tRNAs), messenger RNAs (mRNAs), enhancer RNAs (eRNAs) and a number of noncoding RNAs [7]. Studies have shown that m5C is widespread in human mRNAs [8]. Some researchers have reported that there is a significant difference in m5C mRNA between HCC and adjacent noncancerous liver (ANL) tissues [9]. However, the authors did not conclude that the m5C level was upregulated or downregulated in HCC [9], and the functional role of m5C in the occurrence and development of HCC is still unclear.

Among the eight kinds of human RNA m5C methyltransferases (NSUN1-7 and TRDMT1), only NSUN2 and NSUN6 have been reported to methylate mRNAs [7, 10]. NOP2/SUN domain family member 2 (NSUN2), an RNA methyltransferase, can introduce m5C into cytoplasmic tRNAs and mRNAs and plays indispensable roles in phosphorylation, protein synthesis, cell cycle progression, epidermal differentiation and tumorigenesis [11]. A previous study revealed that NSUN2 is highly expressed in a variety of tumours, and is related to the occurrence and development of tumours [12]. Recently, Sun et al. reported that NSUN2 could promote the proliferation and invasion of HepG2 cells via m5C modification of the long noncoding RNA (lncRNA)-H19 [13]. However, the clinical significance of NSUN2 expression in HCC patients, whether NSUN2 can affect HCC progression through m5C-modified mRNAs, and the underlying mechanism remain largely unknown.

In the present study, we utilised 125 pairs of HCC and corresponding ANL tissues and The Cancer Genome Atlas (TCGA) data to investigate the expression levels of the m5C RNA methyltransferase NSUN2 in HCC. We demonstrated that NSUN2 is upregulated and associated with poor prognosis in HCC patients and promotes HCC growth and metastasis both in vitro and in vivo. In addition, we used m5C RNA immunoprecipitation sequencing (m5C-RIP-seq) to investigate the distribution of m5C methylation in HCC mRNAs. The results revealed that m5C was frequently hypermethylated in HCC and that both upregulated mRNAs and m5C-modified mRNAs were involved mainly in metabolism-associated signalling pathways. Furthermore, we revealed that NSUN2-mediated m5C modification could promote glycolysis and the progression of HCC by upregulating PKM2, which provides a potential prognostic factor and therapeutic target for HCC patients and is bound to constitute a breakthrough in clinical and experimental research.

## Methods

### Patients and samples

In total, 125 pairs of HCC and corresponding ANL tissues (Cohorts 1, 2 and 3), which were obtained from at least 2 cm away from the tumour border and were proven to lack tumour cells by microscopy, were obtained from surgical resections of patients without preoperative treatment at Eastern Hepatobiliary Surgery Hospital (Shanghai, China). Human specimen collection was approved by the Ethics Committee of Eastern Hepatobiliary Surgery Hospital. Written informed consent was obtained from each patient according to the policies of the committee. The resected samples were independently identified by two pathologists. The samples used for RNA- sequencing, RT-qPCR and western blot analysis were freshly frozen, and the samples used for immunohistochemistry (IHC) were formalin-fixed and paraffin-embedded. Among them, 40 pairs (Cohort 1) were used for RT-qPCR and western blotting. Eighty pairs (Cohort 2) were subjected to IHC and the correlations between NSUN2 expression and the clinical characteristics of HCC patients after hepatectomy were analysed. Five pairs (Cohort 3) were used for mRNA m5C dot blotting and m5C-RIP-sequencing. The detailed clinicopathological features are described in Supplementary Table 1.

### Follow-up

The patients in Cohort 2 received check-ups every 2–3 months after surgery during the first 24 months and every 3–6 months thereafter until December 2020. Physicians who were blinded to the study performed the follow-up examinations. Serum AFP levels and abdominal ultrasound examinations were performed every month during the first year after surgery and every 3–6 months thereafter. Computed tomography and/or magnetic resonance imaging were performed every 3–6 months or when recurrence was suspected. The diagnostic of recurrence was based on the diagnosis criteria from the AASLD Practice Guidelines (http://www.aasld.org/practiceguidelines/Documents/Bookmarked%20Practice%20Guidelines/HCCUpdate2010.pdf). Once recurrence was confirmed, further treatment was implemented on the basis of the tumour diameter, the number of tumours, the location of the tumour, and the extent of vessel invasion as well as liver function and performance status. Recurrence-free survival (RFS) was calculated from the date of tumour resection until the detection of tumour recurrence, death from a cause other than HCC, or the last follow-up visit. The overall survival (OS) was defined as the length of time between surgery and either the death of the patient or the last follow-up visit.

### mRNA m5C dot blotting

Using the PolyATtract® mRNA Isolation System III (Z5300), the mRNAs were extracted from the total RNA of five paired HCC and ANL tissues. The mRNA m5C dot blotting assay was performed as previously described with some modifications [4, 14, 15]. Before use, the mRNA samples were heated at 80 °C for 2 min and then quenched at 4 °C for 5 min. One hundred nanograms of mRNA per sample was spotted on a nylon membrane. After being suctioned under negative pressure for ~15 min, the membrane was crosslinked twice under an ultraviolet illuminator and then soaked in blocking buffer for 30 min at room temperature. The membrane was incubated with m5C antibody (Abcam, ab10805) and diluted with blocking buffer at 4 °C overnight. The use of anti-IgG pAb-HRP and chemiluminescence reagents (Omni-ECL™, SQ201) followed the manufacturer’s protocols. The signal was detected with ChemiDoc™ and ChemiDoc MP imaging systems (Bio-Rad).

### m5C-RIP-seq and m5C-RIP-qPCR

mRNA m5C-RIP-seq was performed as previously described [9]. Briefly, the m5C-RIP-seq consisted of RNA extraction, RNA fragmentation, m5C-RIP (Abcam, ab10805), library construction, sequencing and identification of m5C peaks. DiffReps software [16] was used to identify differentially regulated m5C peaks between HCC and ANL tissues. The m5C peaks with *P* ≤ 0.00001 and a fold change ≥2 (or ≤0.5) were considered to be upregulated (or downregulated). The methylation fold enrichment (FE) of each mRNA was subsequently acquired and subjected to log2 conversion. The logFE value was used for clustering in CLUSTER 3.0 software [17]. The detailed information of the antibodies used in this study is listed in Supplementary Table 2.

m5C-RIP-qPCR was conducted as previously described [18]. Briefly, RNA extraction, RNA fragmentation, m5C-RIP (Abcam, ab10805) and RT-qPCR were performed. The primers used to target the m5C peak of PKM2 mRNA are listed in Supplementary Table 3.

### mRNA-Seq analysis

Paired-end reads were harvested from the Illumina HiSeq 4000 sequencer and were quality controlled by Q30. After 3′ adaptor-trimming and removal of low-quality reads via cutadapt software (v1.9.3), the high-quality reads were aligned to the human reference genome (UCSC hg19) with HISAT2 software (v2.0.4). Guided by the Ensembl gtf gene annotation file, Cuffdiff software (v2.2.1, part of Cufflinks) was subsequently used to obtain the FPKMs as the expression profiles of the mRNAs, and the fold change and *p* value were calculated on the basis of the FPKMs. Differentially expressed mRNAs were identified with a fold-change cut-off of 2 and a *p* value cut-off of 0.05.

### Western blot analysis

Radioimmunoprecipitation assay solution (Beyotime, Shanghai, China) was used to harvest lysates from HCC tissues and cells. Protein samples were run on 4%–20% sodium dodecyl sulphate-polyacrylamide gel electrophoresis gels, transferred to polyvinylidene fluoride membranes, and blocked with 5% non-fat milk for 1 h. After incubation with primary antibodies overnight at 4 °C, the membranes were incubated with secondary antibodies for 2 h. Finally, the protein bands were visualised with an enhanced chemiluminescent solution (Beyotime, Shanghai, China) and detected via a chemiluminescence system (Bio-Rad, USA). The primary antibodies used are listed in Supplementary Table 2.

### Immunohistochemistry and tissue microarrays analysis

A tissue microarray (TMA) was constructed as described previously [19]. IHC was performed on the TMA through a two-step immunoperoxidase technique [20]. The immunohistochemical stains were assessed by the histochemistry score [21] by three separate observers who had no knowledge of patient characteristics. The antibodies used are listed in Supplementary Table 2.

### Reverse transcription reaction and RT-qPCR

Total RNA was isolated with TRIzol reagent (Takara, Dalian, China). Complementary DNA (cDNA) was synthesised via a cDNA synthesis kit (Takara, Dalian, China), and RT-qPCR of total RNA was performed via a real-time PCR system (Applied Biosystems, USA). The real-time PCRs were performed in triplicate. ACTB was used as an endogenous control. The relative expression was calculated via the comparative ΔΔCt method. The primer sequences are presented in Supplementary Table 3.

### Cell lines

HepG2, SNU387, Huh7 and Hep3B cells were obtained from the Chinese Academy of Sciences Cell Bank and were authenticated by short tandem repeat profiling. Using the LookOut^®^ Mycoplasma PCR Detection Kit (Sigma-Aldrich), we conformed that all the cell lines used in this study were free of contamination by mycoplasma. The cells were grown in Dulbecco’s modified Eagle’s medium with 10% foetal bovine serum (Gibco BRL). The cells were maintained in an atmosphere of 5% CO_2_ in a humidified 37 °C incubator.

### In vitro cell-behaviour assays

For the migration and invasion assays, Transwell filter chambers (Costar, Corning, NY) were used according to the manufacturer’s instructions. Six random microscopic fields were counted per sample for each group. For the cell-proliferation assays, HCC cells were seeded in 100 μL of growth medium in 96-well plates for various durations. Cell proliferation was evaluated by measuring cell viability with a Cell Counting Kit-8 assay (Dojindo Laboratories, Kumamoto, Japan) according to the manufacturer’s instructions. These experiments were repeated at least three times independently.

### Animal studies

The animal experiments in this study conformed to the Animal Research: Reporting of In Vivo Experiments (ARRIVE) guidelines (http://www.nc3rs.org.uk/arrive-guidelines) and were approved by the Institutional Animal Care and Use Committee of Shanghai University of Traditional Chinese Medicine (Shanghai, China). All the BALB/c nude mice used in this study were male, 5 weeks old, and purchased from the Laboratory Animal Resources of the Chinese Academy of Sciences (Beijing, China). The mice were age-matched and then randomised into different groups. The mice were housed in laminar flow cabinets under specific pathogen-free conditions at room temperature with a 12 h light/dark cycle, with food and water available ad libitum.

Subcutaneous xenografts were established in the bilateral armpits of nude mice via the indicated HCC cells (*n* = 8 for each group). Tumour development was observed twice weekly with a calliper, and the tumour volume was calculated via the following formula: (larger diameter × smaller diameter^2^)/2. The mice were sacrificed 4 weeks later, and pieces of tumour tissue were collected to establish orthotopic implanted models.

For the pulmonary metastatic model, the indicated HCC cells were injected into the tail vein of nude mice (*n* = 6 for each group). Metastases were detected via the IVIS@ Lumina II system (Caliper Life Sciences, Hopkinton, MA) 10 min after the intraperitoneal injection of 4.0 mg of luciferin (Gold Biotech) in 50 μl of saline.

### RNA immunoprecipitation

RNA immunoprecipitation (RIP) experiments were performed via a Magna RIP™ RNA-Binding Protein Immunoprecipitation Kit (Millipore, USA) according to the manufacturer’s instructions.

### Luciferase reporter assay

The PKM-WT or PKM2-Mut plasmid was transfected into NSUN2-overexpressing or NSUN2-silenced HCC cells via Lipofectamine-mediated gene transfer. The relative luciferase activity was normalised to Renilla luciferase activity 48 h after transfection.

### Actinomycin D assay

The actinomycin D assay was performed as previously described [22–24]. HCC cells were seeded equally in 5 wells in 24-well plates (5 × 10^4^ cells per well). Twenty-four hours later, the cells were exposed to actinomycin D (2 µg/ml, Abcam, ab141058) for 0 h, 3 h or 6 h. After that, the cells were harvested, and the relative RNA levels were analysed via RT-qPCR and normalised to the values measured in the mock treatment group (the 0 h group).

### Metabolism measurements

A glucose colorimetric assay kit (K606-100, BioVision, USA) and a lactate colorimetric assay kit II (K627-100, BioVision, USA) were used to determine glucose uptake and lactate production, respectively, according to the manufacturers’ protocols. The extracellular acidification rate (ECAR) and glycolytic proton efflux rate (glycoPER) were examined via a Seahorse XF glycolysis stress test kit (103020-100, Seahorse Bioscience, North Billerica, MA, USA) and a Seahorse XF glycolytic rate assay kit (103344-100, Seahorse Bioscience) according to the manufacturer’s protocol, respectively, and analysed via a Seahorse XFe 96 Extracellular Flux Analyzer (Seahorse Bioscience). Briefly, for the ECAR, glucose, oligomycin (an oxidative phosphorylation inhibitor), and 2-deoxyglucose (2-DG, a glycolytic inhibitor) were sequentially added to the wells of a Seahorse XF96 cell culture microplate at the indicated time points. For glycoPER, rotenone + antimycin A (Rot/AA) and 2-DG were sequentially added to the wells of a Seahorse XF96 cell culture microplate at the indicated time points.

### Statistical analysis

The investigators were blinded to group allocation for animal studies. All the statistical analyses were performed via SPSS (v.23.0) and GraphPad Prism (v.8). Differences between groups were examined by Student’s *t*-test, the Wilcoxon signed-rank test, the Mann–Whitney *U* test or the chi-square test. Correlations were measured via Spearman correlation analysis. Survival curves were generated via the Kaplan–Meier method, and differences were assessed via the log-rank test. Statistical significance was indicated by *p* values less than 0.05. ^*^P < 0.05, ^**^P < 0.01, ^***^P < 0.001.

## Results

### NSUN2 is upregulated and associated with poor prognosis in HCC patients after hepatectomy

**Fig. 1:**
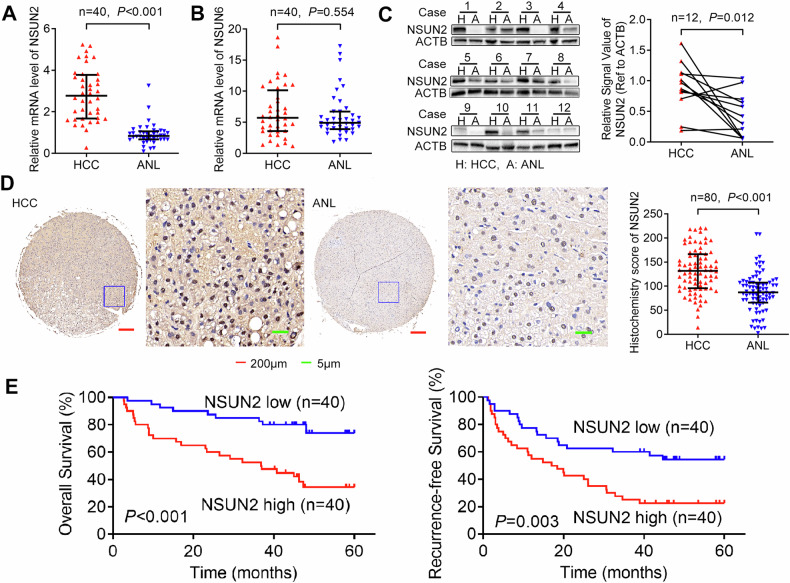
Upregulated NSUN2 expression predicts poor prognosis in patients with HCC. The expression of NSUN2 (**A**) and NSUN6 (**B**) mRNAs was analysed via RT-qPCR. The Wilcoxon signed-rank test was used. **C** NSUN2 protein expression was analysed via western blot analysis. Student’s *t*-test was used. **D** IHC staining of NSUN2. (Right) Histochemistry score of NSUN2 in 80-paired HCC tissues and ANL tissues. (Left) Representative samples. **E** Kaplan–Meier analysis of the overall survival and recurrence-free survival of 80 HCC patients. ANL adjacent noncancerous liver, HCC hepatocellular carcinoma, RT-qPCR quantitative reverse transcription PCR.

To determine the role of mRNA m5C modification in HCC, we evaluated the expression levels of two major mRNA m5C methyltransferases, NSUN2 and NSUN6 (the only two known RNA m5C methyltransferases that methylate mRNA [7, 10]), in 40-paired HCC and ANL tissues via RT-qPCR (Cohort 1). The results revealed that the mRNA level of NSUN2 was increased in HCC (Fig. 1A), whereas that of NSUN6 was no significantly different (Fig. 1B). Furthermore, western blotting of 12 paired HCC and ANL tissues (randomly selected from Cohort 1) revealed that the protein level of NSUN2 was also upregulated in HCC (Fig. 1C).

To determine the clinical importance of high-NSUN2 expression in HCC, we detected the expression of NSUN2 in another cohort of 80 HCC patients (Cohort 2) with prognostic data via IHC (Fig. 1D). Moreover, NSUN2 protein was overexpressed in HCC. According to the median IHC score of NSUN2 in HCC tissues, we divided the 80 HCC patients into a high-NSUN2-expressing group and a low-NSUN2-expressing group. We found that higher expression of NSUN2 was correlated with the presence of pathological satellites (*P* = 0.025), larger tumour size (*P* = 0.013) and microvascular invasion (*P* = 0.041) (Table 1). These findings indicate that NSUN2 is related to HCC growth and metastasis. Furthermore, Kaplan–Meier’s survival curves revealed that higher expression of NSUN2 was associated with lower OS (*P* < 0.001) and lower RFS (*P* = 0.003) in HCC patients after hepatectomy (Fig. 1E). In addition, according to the TCGA database in GEPIA2 [25], higher NSUN2 mRNA levels also predict poorer OS in HCC patients (*P* = 0.023, Supplementary Fig. 1).

**Table 1:** Clinical characteristics of 80 HCC patients according to NSUN2 expression level.

Variable	NSUN2		*P* value
	High	Low	
All cases	40	40	
Age, years, >50: ≤ 50	19:21	17:23	0.653
Gender, male: female	34:6	27:13	0.066
HBsAg, positive: negative	36:4	39:1	0.356
Liver cirrhosis, with: without	28:12	26:14	0.633
AFP, μg/L, >20: ≤ 20	29:11	27:13	0.626
Pathological satellite, present: absent	24:16	14:26	0.025*
Tumour number, multiple: solitary	9:31	5:35	0.239
Edmondson’s grade, III + IV: I + II	35:5	32:8	0.363
Tumour size, cm, >5: ≤ 5	28:12	17:23	0.013*
Microvascular invasion, present: absent	21:19	12:28	0.041*
Encapsulation, incomplete: complete	30:10	22:18	0.061
TNM stage, II + III:I	10:30	6:34	0.264
BCLC stage, B + C:0 + A	9:31	4:36	0.130

χ^2^ test was used to test the association between two categorical variables.

*Statistically significant.

The *P*-values of these three items are less than 0.05, so they are bolded.

### NSUN2 promotes HCC growth and metastasis in vitro and in vivo

We then explored whether NSUN2 affects HCC progression. For verification, we assessed the expression of NSUN2 mRNA in multiple HCC cell lines (Supplementary Fig. 2A). Using lentivirus, NSUN2 was stably overexpressed in the HCC cell lines HepG2 and SNU387 (Supplementary Fig. 2B, C). We also stably silenced NSUN2 in the HCC cell lines Hep3B and Huh7 via lentivirus-mediated short hairpin RNAs (NSUN2-sh1, NSUN2-sh2 and NSUN2-sh3) (Supplementary Fig. 2B, C). Given that NSUN2-sh2 and NSUN2-sh3 were more effective than NSUN2-sh1 was, NSUN2-sh2 and NSUN2-sh3 were used for further study.

**Fig. 2:**
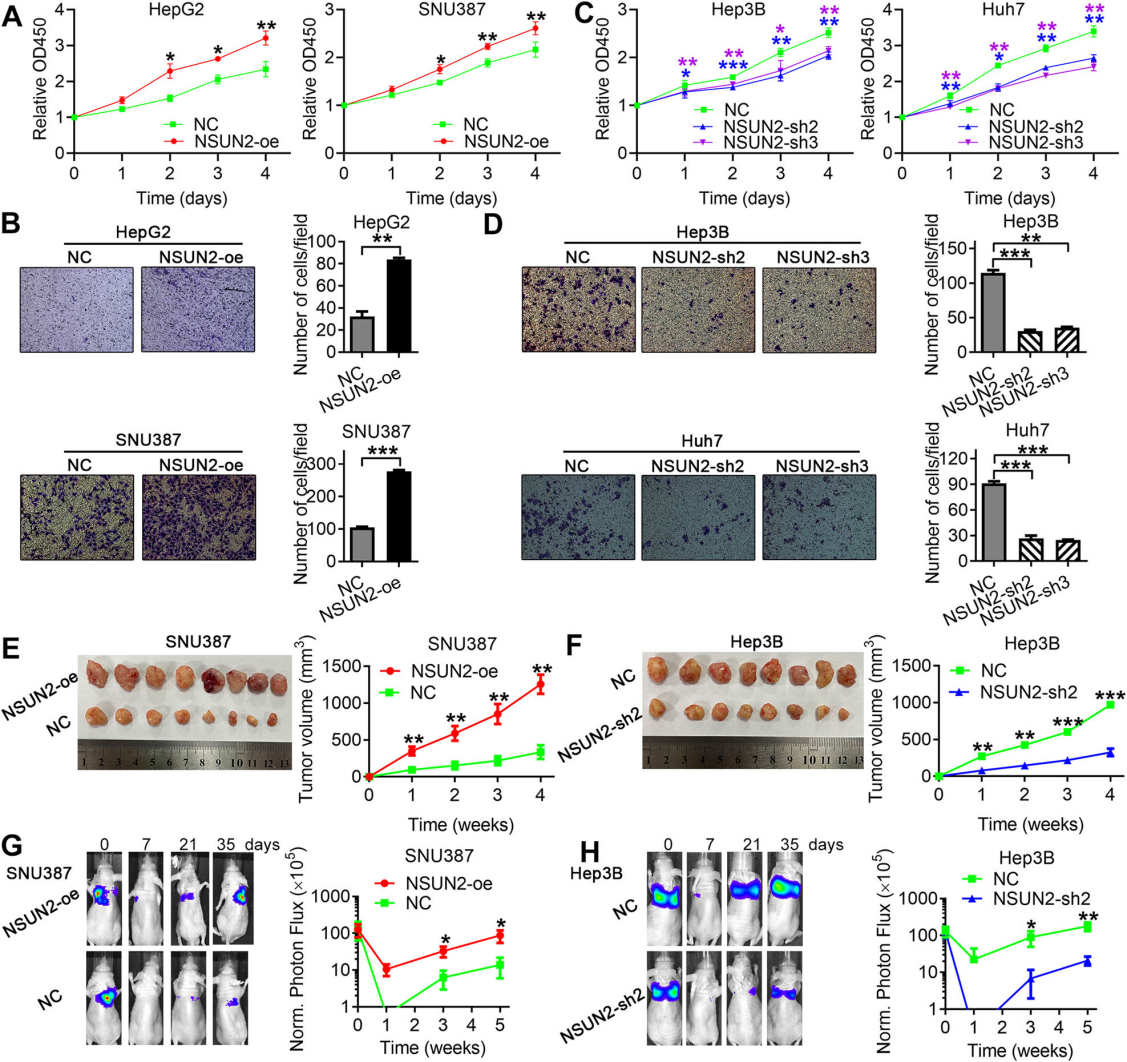
NSUN2 promotes HCC growth and metastasis in vitro and in vivo. **A** Cell Counting Kit-8 assays revealed that NSUN2 overexpression promoted the growth of HCC cells. **B** Transwell migration assays revealed that NSUN2 overexpression promoted the migration of HCC cells. **C** Cell Counting Kit-8 assay results showing that NSUN2 knockdown inhibited the growth of HCC cells. **D** Transwell migration assays revealed that NSUN2 knockdown inhibited the migration of HCC cells. **E**, **F** Subcutaneous xenografts from the indicated HCC cells were excised from nude mice, and tumour weights and tumour growth curves were generated. Eight mice were included in each group. **G**, **H** (Right) Luciferase signal intensities of mice over time after tail vein injection of the indicated HCC cells. (Left) Representative images. There were six mice in each group. For (**A**–**D**), the data are presented as the means ± SDs; *n* = 3. Student’s *t*-test was used. For (**E**–**H**), the data are presented as the means ± SEM. The Mann–Whitney *U* test was used.

CCK-8 and Transwell assays revealed that forced expression of NSUN2 promoted the growth and metastasis of HCC (Fig. 2A, B) and that NSUN2 depletion inhibited the growth and metastasis of HCC (Fig. 2C, D).

To explore the role of NSUN2 in HCC growth in vivo, the NSUN2-overexpressing SNU387 cells and the corresponding negative control and the NSUN2-silenced (NSUN2-sh2) Hep3B cells and the corresponding negative control were subcutaneously injected into nude mice. The tumour volume was evaluated every week until the nude mice were sacrificed after 4 weeks. NSUN2 overexpression significantly promoted the growth of SNU387 cells, and NSUN2 knockdown inhibited the growth of Hep3B cells (Fig. 2E, F).

Furthermore, to identify the function of NSUN2 in HCC metastasis in vivo, we established a lung metastasis model by injecting NSUN2-overexpressing or NSUN2-silenced HCC cells into the tail veins of nude mice. Using an in vivo imaging system, the metastatic process of the HCC cells in the lung was monitored for 5 weeks. As a result, the upregulation of NSUN2 increased the lung metastasis of HCC cells, whereas the downregulation of NSUN2 decreased lung metastasis (Fig. 2G, H).

Overall, NSUN2 could induce HCC growth and metastasis in vitro and in vivo.

### NSUN2-mediated m5C hypermethylation promotes metabolism in HCC

**Fig. 3:**
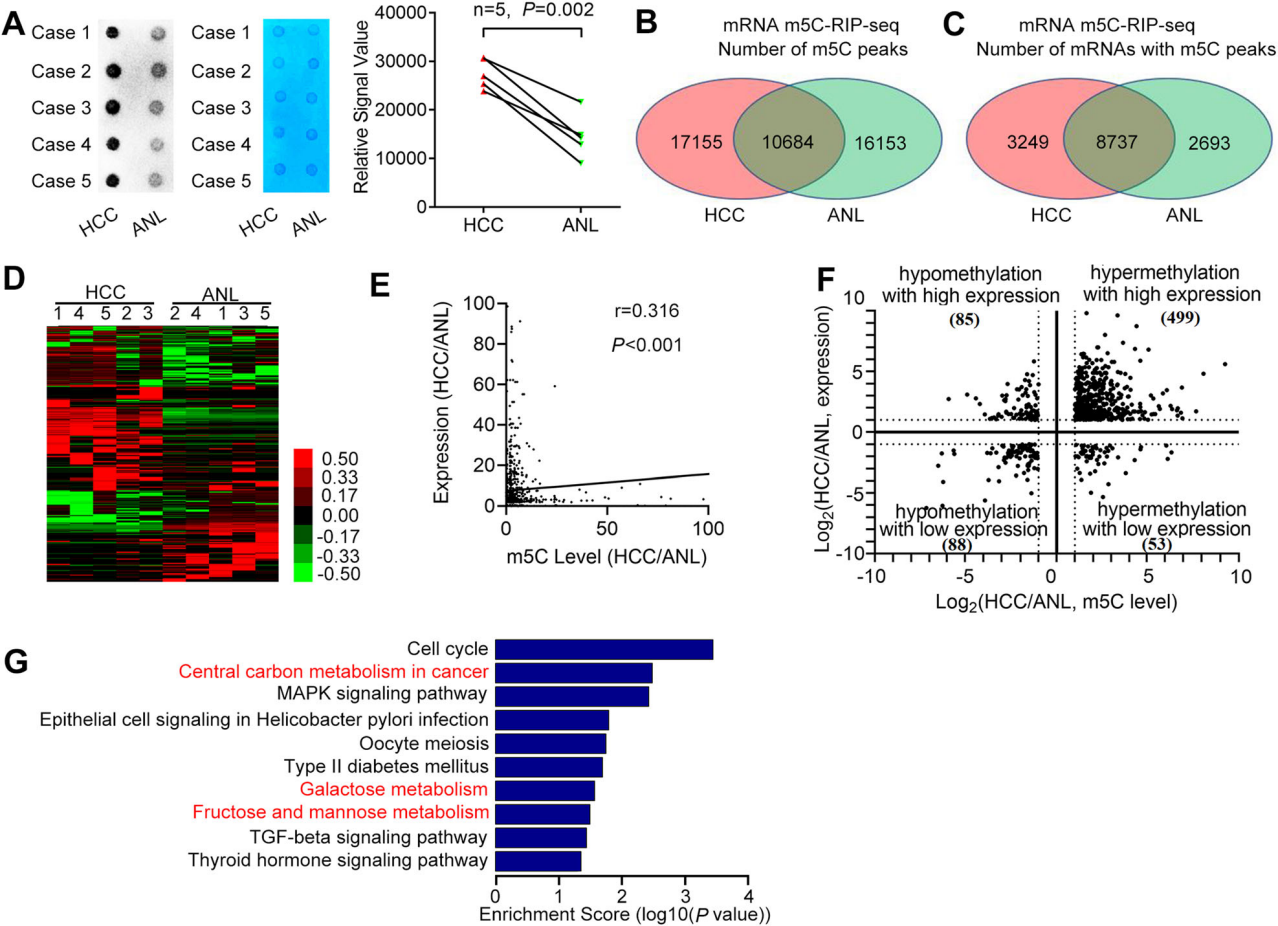
Overview of mRNA m5C in human HCC and ANL tissues. **A** Dot blot analysis of 5-methylcytosine (m5C) in mRNAs from five paired HCC and ANL tissues (50 ng each). Student’s *t*-test was used. **B** The numbers of m5C peaks in HCC and ANL tissues. **C** The numbers of mRNAs with m5C peaks in HCC and ANL tissues. **D** Cluster analysis of the differential m5C methylation in five paired HCC and ANL tissues. **E** Correlation analysis between the m5C level and the transcript level. Spearman correlation was used. **F** Distribution of mRNAs whose m5C methylation and gene expression levels significantly changed. **G** KEGG pathway enrichment for the mRNAs with both upregulated expression and m5C levels. ANL adjacent noncancerous liver, HCC hepatocellular carcinoma.

We next explored whether the oncogenic role of NSUN2 is m5C-dependent in HCC. First, to detect the global mRNA m5C level in human HCC, we performed m5C dot blotting in five paired HCC and ANL tissues (Cohort 3) (Fig. 3A). The global mRNA m5C level in HCC was greater than that in ANL tissues. Furthermore, m5C-RIP-seq of mRNAs from the five pairs of HCC and ANL tissues was performed to elucidate the transcriptomic m5C profile of HCC. The m5C peaks in each sample are shown in Supplementary Tables 4 and 5. Using BEDTools, we identified 27839 unique m5C peaks in 11986 mRNAs in HCC tissues (Supplementary Table 6) and 26837 unique m5C peaks in 11430 mRNAs in ANL tissues (Supplementary Table 7). Among them, 10684 m5C peaks in 8737 mRNAs were detected in both HCC and ANL tissues (Fig. 3B, C). The m5C levels of mRNAs in HCC and ANL were compared, and 3156 methylation peaks in 2696 mRNAs in HCC were identified as upregulated (Supplementary Table 8), whereas 1998 methylation peaks in 1756 mRNAs were detected as downregulated (Supplementary Table 9) (Fig. 3D). Moreover, mRNA-sequencing (mRNA-Seq) of the same cohort revealed 3137 upregulated mRNAs (Supplementary Table 10) and 542 downregulated mRNAs (Supplementary Table 11) in HCC compared with ANL. Taken together, these findings indicate that m5C in mRNAs is frequently hypermethylated in HCC.

The joint analysis of the mRNA m5C-RIP-seq and mRNA-Seq data revealed that the expression of mRNAs in HCC (normalised to that in ANL tissues) was slightly positively correlated with (Spearman *r* = 0.316, *P* < 0.001) their m5C levels (Fig. 3E). Moreover, among the 3156 upregulated m5C peaks, the expression of 499 (15.81%) corresponding mRNAs was upregulated in HCC, whereas that of only 53 (1.68%) was downregulated in HCC (Fig. 3F). Among the 1998 downregulated m5C peaks, 85 (4.25%) corresponding mRNAs were upregulated in HCC, and 88 (4.40%) were downregulated in HCC. These results indicate that m5C hypermethylation was positively correlated with mRNA overexpression in HCC.

KEGG pathway analyses revealed that the mRNAs with both upregulated expression and m5C levels were enriched in ten pathways (Fig. 3G). Interestingly, central carbon metabolism in cancer (HK2, MAPK3, MYC, PFKP, PKM2, SLC16A3), galactose metabolism (B4GALT2, HK2, PFKP) and fructose and mannose metabolism (HK2, PFKFB4, PFKP) are associated with metabolism.

Overall, m5C hypermethylation is associated with mRNA overexpression and metabolism in HCC.

### PKM2 is the main target of NSUN2-mediated m5C modification to promote metabolism

**Fig. 4:**
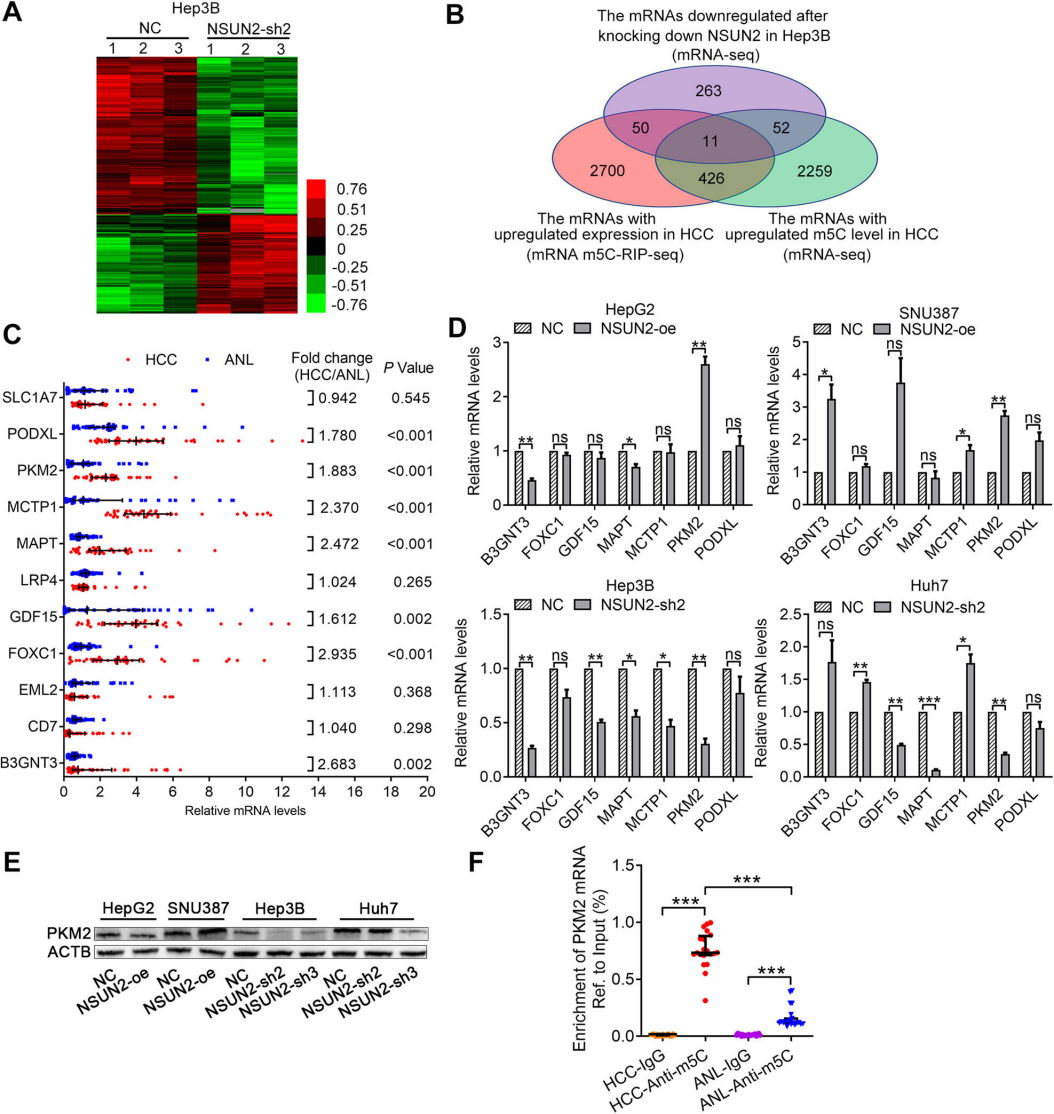
PKM2 mRNA is the main target of NSUN2. **A** Cluster analysis of the differentially expressed mRNAs after NSUN2 was knocked down in Hep3B cells. **B** Screening of the potential target mRNAs of NSUN2 in human HCC. **C** RT-qPCR results showing the mRNA levels of the 11 candidate genes in 40-paired HCC and ANL tissues. **D** RT-qPCR analysis of the mRNA levels of the 7 candidate genes in HCC cells. **E** The protein expression of PKM2 in HCC cells was analysed by western blot analysis. **F** The enrichment of PKM2 mRNA in 20 paired HCC and ANL tissues was identified via m5C-RIP-qPCR using anti-IgG or anti-m5C antibodies. For (**C**, **F**), the Wilcoxon signed-rank test was used. For (**D**), Student’s *t*-test was used.

To identify the target mRNAs of NSUN2 in human HCC, we performed mRNA-sequencing of Hep3B-NSUN2-sh2 cells and the corresponding negative control. After NSUN2 was knocked down, 236 genes were upregulated, and 376 genes were downregulated (Fig. 4A, Supplementary Table 12). We then overlapped the mRNAs with upregulated expression in HCC, the mRNAs with upregulated m5C levels in HCC, and the mRNAs whose expression was downregulated after NSUN2 was knocked down in Hep3B cells. A Venn diagram revealed that eleven mRNAs (B3GNT3, CD7, EML2, FOXC1, GDF15, LRP4, MAPT, MCTP1, PKM2, PODXL and SLC1A7) met these criteria (Fig. 4B, C). We then detected the expression of these eleven mRNAs in 40-paired HCC and ANL tissues and found that seven of them were upregulated in HCC (Fig. 4C). Furthermore, we detected the expression of these seven mRNAs in HCC cell lines and found that only PKM2 was upregulated after NSUN2 was overexpressed in both HepG2 and SNU387, cells but downregulated after NSUN2 was silenced in both Hep3B and Huh7 cells (Fig. 4D, E).

According to the m5C-RIP-seq results, the upregulated m5C peak of PKM2 mRNA is located at its 3′-UTR (chr15:72491753-72491855, hg19). m5C-RIP-qPCR targeting this peak validated these results (Fig. 4F).

Therefore, we assumed that PKM2 mRNA is the main target of NSUN2.

### NSUN2 stabilises PKM2 mRNA by increasing its m5C level

**Fig. 5:**
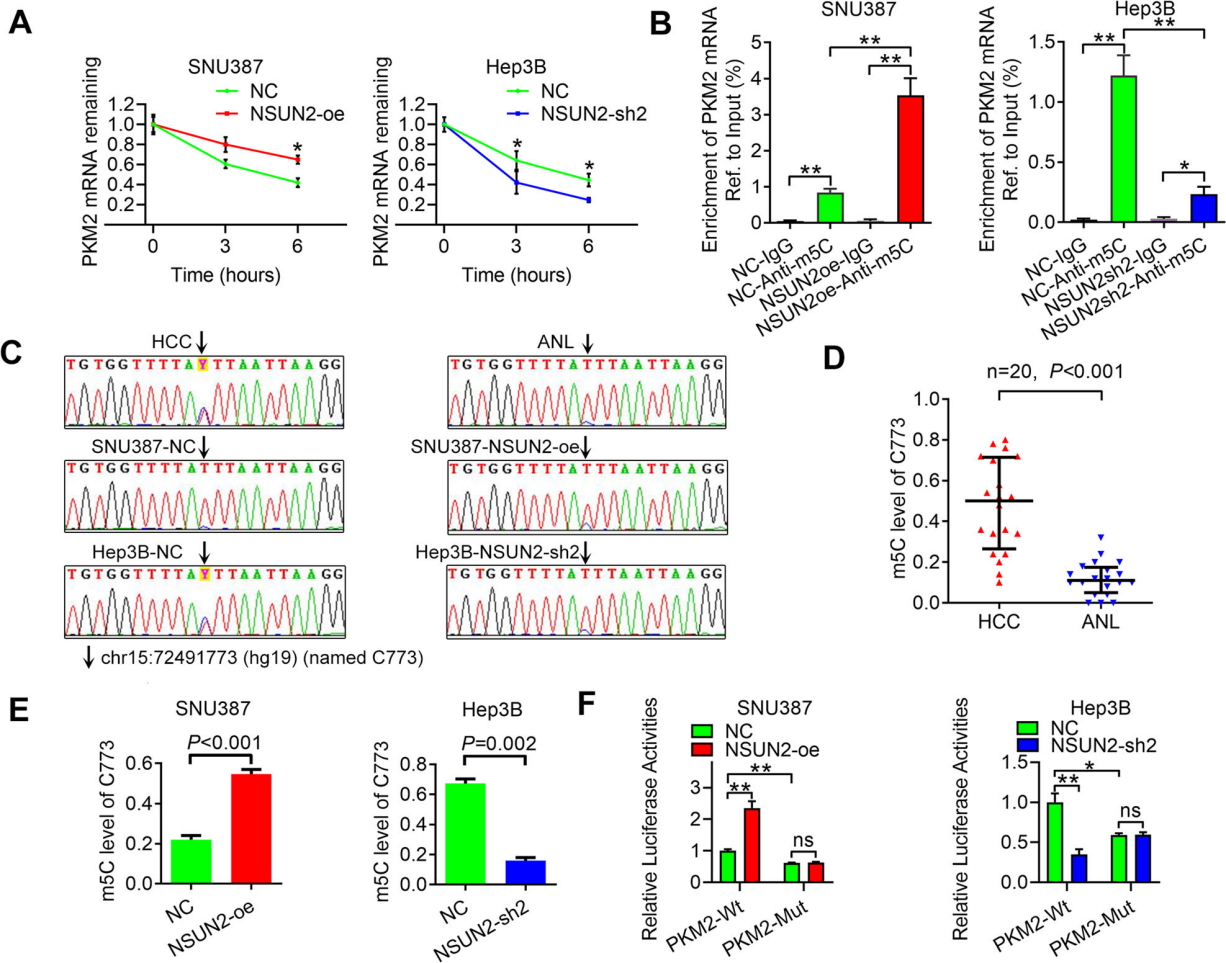
NSUN2 could stabilise PKM2 mRNA by increasing its m5C level. **A** The relative RNA level of PKM2 was analysed by RT-qPCR after treatment with actinomycin D at the indicated time points in HCC cells. **B** Enrichment of PKM2 mRNA in the indicated HCC cells was identified via m5C-RIP-qPCR using anti-IgG or anti-m5C antibodies. **C** Sanger sequencing was performed after bisulfite- PCR. Representative sequencing chromatograms showing the m5C site (chr15:72491773 (hg19) (named C773)) of PKM2 mRNA. **D**, **E** The m5C level of C773 in the indicated samples is shown. **F** Relative luciferase activity of the luciferase reporter gene with the wild-type C773 site (PKM2-Wt) or the mutant C733 site (PKM2-Mut) in the indicated HCC cells. For (**A**–**C**, **E**, **F**), Student’s *t*-test was used. For (**D**), the Wilcoxon signed-rank test was used.

Next, we investigated whether NSUN2 could induce the expression of PKM2 by stabilising PKM2 mRNA through increasing its m5C level. We found that overexpressing NSUN2 slowed the degradation of PKM2 mRNA, whereas silencing NSUN2 had the opposite effect (Fig. 5A). These findings confirmed that NSUN2 could stabilise PKM2 mRNA. Furthermore, m5C-RIP-qPCR revealed that the m5C level of PKM2 mRNA was increased after NSUN2 was overexpressed in SNU387 cells but decreased upon NSUN2 knockdown in Hep3B cells (Fig. 5B).

To detect the exact m5C site of PKM2, we performed bisulfite-PCR [13] by using primers targeting the m5C peak of PKM2 and cDNA template reverse transcribed from bisulfite converted RNA. Representative Sanger sequencing chromatograms revealed that the signal at chr15:72491773 (hg19) (named C773) was mixed with cytosine (‘C’, blue) and thymine (‘T’, red), indicating that m5C was methylated in both the HCC tissues and the cell lines (Fig. 5C).

Using a previously described method [26], we calculated the m5C level in each sample. For example, PCR products from SNU387-NC were subcloned and inserted into the T-easy vector, and 50 clones were randomly selected and sequenced. Among them, 9 clones were detected as ‘C’, whereas 41 clones were detected as ‘T’. Therefore, the m5C level of C773 in SNU387-NC was calculated as 18% (9/50). Each sample was detected three times. Using this method, the m5C level of C773 in other samples was also detected (Fig. 5D–F). The m5C level in HCC tissues was greater than that in ANL tissues (Fig. 5D). Furthermore, the m5C level increased after NSUN2 was overexpressed in SNU387 cells but decreased after NSUN2 was knocked down in Hep3B cells (Fig. 5E).

To validate the role of C773 in regulating the expression of PKM2, we performed a luciferase reporter assay using a plasmid with the wild-type 3′-UTR of PKM2 mRNA (PKM2-WT) or with a mutated C773 (PKM2-Mut). As expected, forced expression of NSUN2 increased the luciferase activity of PKM2-WT but not that of PKM2-Mut (Fig. 5F), while silencing NSUN2 inhibited it, indicating that the regulatory effect of NSUN2 on PKM2 was dependent on the m5C site C773.

In summary, NSUN2 could stabilise PKM2 mRNA by increasing the m5C level of the m5C site C773 in the 3′-UTR of PKM2 mRNA.

### NSUN2 promotes HCC glycolysis and progression by upregulating PKM2

**Fig. 6:**
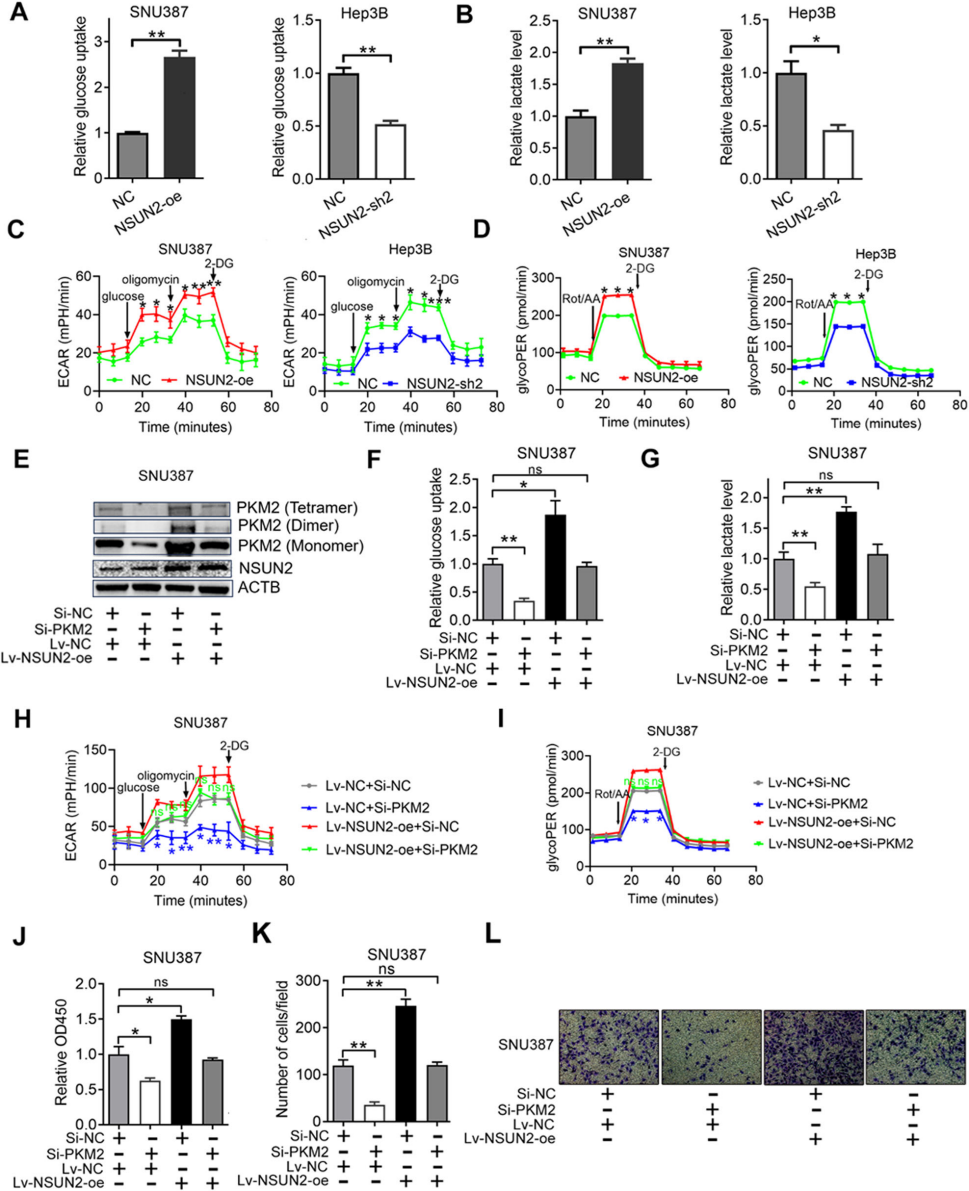
NSUN2 promotes HCC glycolysis and progression by upregulating PKM2. **A**, **F** The relative glucose uptake of HCC cells was measured with a glucose colorimetric assay kit (K606-100, BioVision, USA). For (**B**, **G**), the relative lactate level of HCC cells was measured with a lactate colorimetric assay kit II (K627-100, BioVision, USA). For (**C**, **H**), the extracellular acidification rate (ECAR) of HCC cells was analysed via a Seahorse XF96 instrument (Seahorse Bioscience, USA). 2-DG, 2-deoxyglucose. For (**D**, **I**), the glycolytic proton efflux rate (glycoPER) of HCC cells was analysed via a Seahorse XF96 instrument (Seahorse Bioscience, USA). Rot/AA, rotenone + antimycin A; 2-DG, 2-deoxyglucose. The results were normalised per 10,000 cells. **E** Western blot analysis of the expression of the PKM2 monomer, dimer, tetramer and NSUN2 in SNU387 cells. **J** The growth ability of SNU387 cells was measured via a Cell Counting Kit-8 assay on the third day after co-transfection. **K** The ability of SNU387 cells to metastasize was measured via a Transwell assay. **L** Representative images for (**K**).

The Warburg effect is characterised by increased glucose uptake and lactate production [27]. To test whether NSUN2 could promote the progression of HCC by enhancing the PKM2-mediated Warburg effect, we detected glucose uptake and lactate production after NSUN2 was overexpressed and knocked down. We found that overexpressing NSUN2 significantly increased glucose uptake and lactate production, while silencing NSUN2 decreased glucose uptake and lactate production in HCC cells (Fig. 6A, B). Importantly, NSUN2 overexpression increased the ECAR (reflecting overall glycolytic flux), whereas NSUN2 silencing decreased the ECAR of HCC cells (Fig. 6C). GlycoPER (subtracting CO2-dependent acidification related to respiratory activity) was also performed to examine the glycolytic capacities of the cells after NSUN2 was overexpressed and silenced. The results showed that overexpressing NSUN2 resulted in a significant increase in glycoPER in HCC cells. Furthermore, silencing NSUN2 resulted in a reduction in the glycoPER in HCC cells (Fig. 6D).

We next investigated whether the effect of NSUN2 on HCC was mediated by PKM2. First, we found that siRNA targeting PKM2 could downregulate the expression of the PKM2 tetramer, dimer, and monomer (the expression ratios of tetramer, dimer and monomer did not change), while overexpressing NSUN2 simultaneously could retard this effect (Fig. 6E). Second, we found that silencing PKM2 inhibited glucose uptake and lactate production, and this effect was blocked by overexpressing NSUN2 (Fig. 6F, G). Furthermore, silencing PKM2 decreased the ECAR and glycoPER, whereas overexpressing NSUN2 simultaneously retarded this effect (Fig. 6H, I). In addition, knocking down PKM2 inhibited the growth and invasion of HCC cells, and this effect was blocked by overexpressing NSUN2 (Fig. 6J–L).

In summary, NSUN2 promotes HCC glycolysis and progression by upregulating PKM2.

## Discussion

**Fig. 7:**
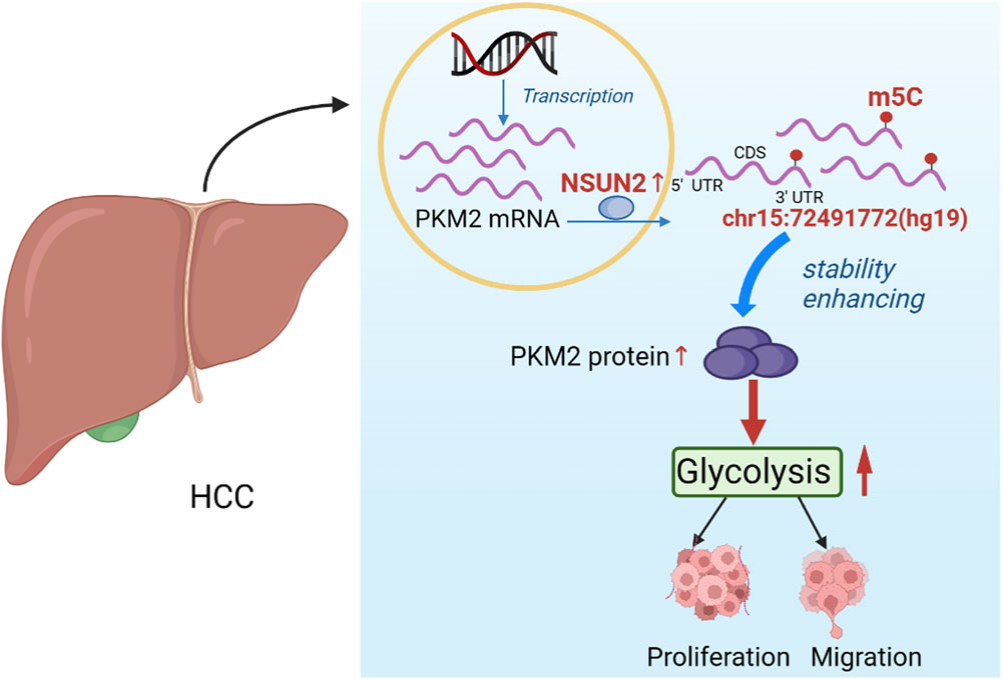
Schematic diagram depicting the proposed mechanisms of NSUN2-mediated m5C modulation of PKM2 in HCC. NSUN2 is upregulated in HCC, and upregulated NSUN2 can stabilise PKM2 mRNA by increasing the m5C level of the m5C site C773 at the 3′-UTR of PKM2 mRNA, thus contributing to increased glycolysis and subsequent HCC progression and migration. Created via BioRender software.

HCC is the most common type of primary liver cancer, accounting for 85–90% of all cases, and has a high mortality rate. Currently, progress has been made in the development of methods for the diagnosis and treatment of HCC, but the prevention of HCC recurrence or metastasis remains a challenge because the understanding of its complex molecular pathogenesis is insufficient. Therefore, new effective molecular diagnostics and therapeutic targets are urgently needed. In the present study, we investigated not only the effect of NSUN2-mediated m5C modification on HCC growth, but also the correlation of this m5C modification with the clinical prognosis of patients with HCC, as well as the possible molecular mechanism involved. We found that the upregulation of NSUN2 was associated with elevated mRNA m5C levels in HCC and was associated with poor prognosis in HCC patients after hepatectomy. We demonstrated for the first time that PKM2 is a target gene of NSUN2-mediated m5C modification and that NSUN2 can promote HCC progression by enhancing PKM2-mediated glycolysis (Fig. 7).

m5C is an RNA modification that has attracted increasing attention. It can dynamically regulate a variety of biological functions through its regulators and plays an important role in a variety of cancers. Our recent study revealed that the m5C RNA methyltransferase NSUN5 promoted the development of HCC [28]. NSUN2, also known as MYC-induced SUN-domain-containing protein, is a methyltransferase that catalyses 5-methylcytosine formation in the mRNAs of mammals. Accumulating evidence has shown that NSUN2-mediated mRNA m5C modification plays a vital role in various cancers, such as gastric cancer [29], oesophageal squamous cell carcinoma [30], and bladder cancer [31]. In the present study, obviously increased NSUN2 was verified in our 40-paired fresh HCC samples and their corresponding adjacent noncancerous liver tissues via qPCR and another independent 80-paired HCC samples and their corresponding adjacent noncancerous liver tissues via TMA IHC staining. Importantly, we found that higher expression of NSUN2 was strongly correlated with lower OS and lower RFS in HCC patients after hepatectomy in both the TCGA database and our HCC verification cohort. NSUN2 expression, along with several clinicopathological indicators, such as pathological satellites, larger tumour size and the presence of microvascular invasion, are important prognostic factors for predicting the OS rate and RFS rate of HCC patients. Furthermore, the results of functional studies from in vitro CCK-8 and transwell assays and in vivo subcutaneous xenografts and pulmonary metastatic models in nude mice revealed that while NSUN2 overexpression induced the growth and metastasis of HCC, NSUN2 silencing inhibited growth and metastasis. Taken together, these data indicate that the NSUN2 level is a potential marker that is strongly correlated with the prognosis of HCC patients. Therefore, further study of the mechanism of NSUN2 in HCC is highly important.

To further explore whether the oncogenic role of NSUN2 is m5C-dependent in HCC, we examined the mRNA m5C level in human HCC and found that the m5C level in HCC tissues was greater than that in ANL tissues. To explore m5C modifications in HCC, we subsequently performed RNA m5C-RIP-seq to identify candidate RNAs associated with NSUN2. RNA m5C-RIP-seq was performed to identify m5C peaks on mRNAs [9], lncRNAs [32] and circular RNAs (circRNAs) [33] in human HCC and adjacent tissues. However, the sequencing results were not validated experimentally. Sun et al. reported that NSUN2 could promote the proliferation and invasion of HepG2 cells via m5C modification of the long lncRNA-H19 [13]. However, the clinical significance of NSUN2 expression in HCC and whether NSUN2 affects the progression of HCC through m5C-modified mRNAs remain largely unknown. In this study, we found that mRNA m5C is frequently hypermethylated in HCC. As m5C has been reported to stabilise mRNAs in bladder cancer [31], we sought to analyse whether this approach was applied to HCC. To test this hypothesis, we analysed m5C methylation and transcriptome data and found that the expression of mRNAs in HCC was slightly positively correlated with m5C levels. Interestingly, KEGG pathway analyses revealed that the mRNAs with both upregulated expression and m5C levels were correlated with metabolism. The liver is the largest metabolic organ in the human body, and metabolic abnormalities are closely related to the occurrence and development of HCC. Therefore, we hypothesised that NSUN2 may be involved in the occurrence and development of HCC by regulating the m5C modification of metabolism-related genes. To identify the target mRNAs of NSUN2 in human HCC, we overlapped the mRNAs with upregulated expression in HCC, the mRNAs with upregulated m5C levels in HCC and the mRNAs whose expression was downregulated after NSUN2 was knocked down in Hep3B cells. The results revealed that eleven mRNAs met this criterion, and the expression of PKM2, a key enzyme in glucose metabolism, was found to be the most consistent with this criterion via PCR verification.

PKM2, a terminal enzyme in the glycolytic pathway [34], is upregulated and associated with poor prognosis in HCC patients [35]. Several studies have reported how PKM2 is regulated in HCC. For example, the circRNA circMAT2B can upregulate PKM2 mRNA expression by sponging miR-338-3p [36]. The lncRNA LNCAROD can bind to SRSF3 to induce PKM switching towards PKM2 and maintain PKM2 level by sponging miR-145-5p [37]. ZFP91 can inhibit PKM splicing, resulting in lower levels of the PKM2 isoform and higher levels of the PKM1 isoform [27]. However, whether PKM2 can be regulated by RNA methylation remains unknown. In this study, we identified an m5C RNA methylation site (chr15:72491773 (hg19)) (named C773) at the 3′-UTR of PKM2 mRNA via m5C-RIP-seq, and m5C-RIP-qPCR targeting this peak validated the result. We tested whether NSUN2 influences the expression of PKM2 by stabilising PKM2 mRNA through increasing its m5C level. Through actinomycin D and m5C-RIP-qPCR assays, we found that overexpressing NSUN2 could retard the degradation of PKM2 mRNA and increase the m5C level of PKM2 mRNA, whereas silencing NSUN2 had the opposite effect. Moreover, a luciferase reporter assay indicated that the regulatory effect of NSUN2 on PKM2 was dependent on the m5C site C773. Taken together, these data, for the first time, show that NSUN2 can stabilise PKM2 mRNA by increasing the m5C level of the m5C site C773 in the 3′-UTR of PKM2 mRNA. Glucose metabolic reprogramming, characterised by increased glycolysis despite the presence of oxygen (Warburg effect), provides essential metabolic intermediates for biosynthesis, thus promoting the progression of cancers, including HCC [38, 39]. PKM2 can enhance the Warburg effect by catalysing the conversion of phosphoenolpyruvate and ADP to pyruvate and ATP, thus promoting the occurrence and metastasis of HCC [40, 41]. These findings prompted us to investigate whether NSUN2 could promote the progression of HCC by enhancing the PKM2-mediated Warburg effect. By analysing the metabolic phenotype of HCC cells, we found that NSUN2 overexpression increased glucose uptake, lactate production, the ECAR and glycoPER. These findings revealed the underlying mechanism by which NSUN2-mediated PKM2 regulates HCC glycolysis. By performing further rescue assays, we subsequently confirmed that NSUN2 could promote HCC glycolysis and progression by upregulating PKM2.

## Conclusions

In summary, our study revealed the critical role of NSUN2 in HCC progression and confirmed that this role is dependent on its m5C modification. The influence of NSUN2 on promoting HCC growth and metastasis was further demonstrated in vitro and in vivo. Mechanistically, NSUN2-mediated m5C modification promotes glycolysis and the progression of HCC by stabilising PKM2 mRNA. These findings suggest that NSUN2-mediated m5C modification plays an important role in HCC and provide potential prognostic factors and therapeutic targets for HCC patients.

## Supplementary information

Full and uncropped western blots

Supplementary Figures 1 and 2 and Supplementary Tables 1–3

Supplementary Tables 4–12

## Data Availability

m5C-RIP-Seq data that support the findings of this study have been deposited in the Gene Expression Omnibus database under accession number GSE278763. RNA-seq data are available from the NCBI Sequence Read Archive (SRA) database (BioProject Accession: PRJNA1181112, SUB14831975). All other data that support the findings of this study are available from the corresponding authors upon reasonable request.
