# Supplementary material for: The RNA M5C methyltransferase NSUN2 promotes progression of hepatocellular carcinoma by enhancing PKM2-mediated glycolysis
Source: Cell Death Dis. 2025 Feb 9;16(1):82. doi: 10.1038/s41419-025-07414-5 (PMC11808121; doi:10.1038/s41419-025-07414-5)
Supplement: Supplementary file 1 — Full and uncropped western blots [file 41419_2025_7414_MOESM1_ESM.docx]

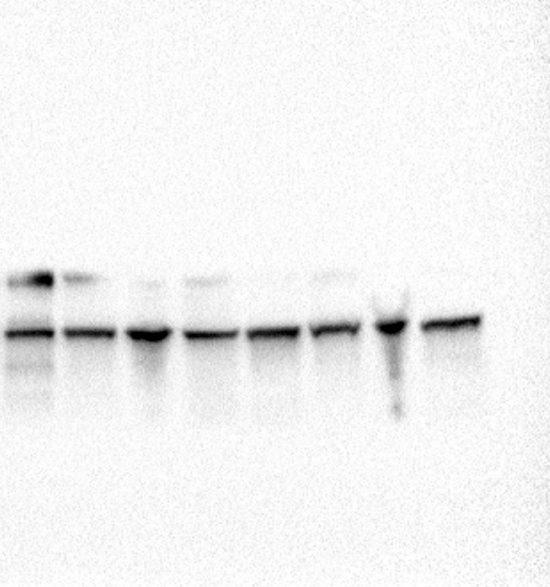

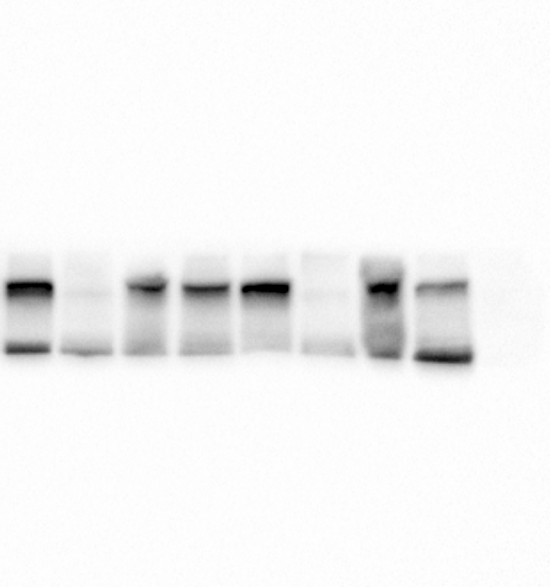


Figure 1C ACTB-1 Figure 1C NSUN2-1


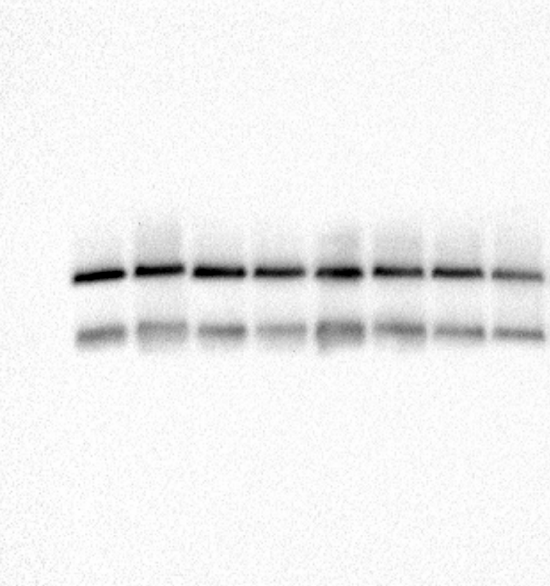

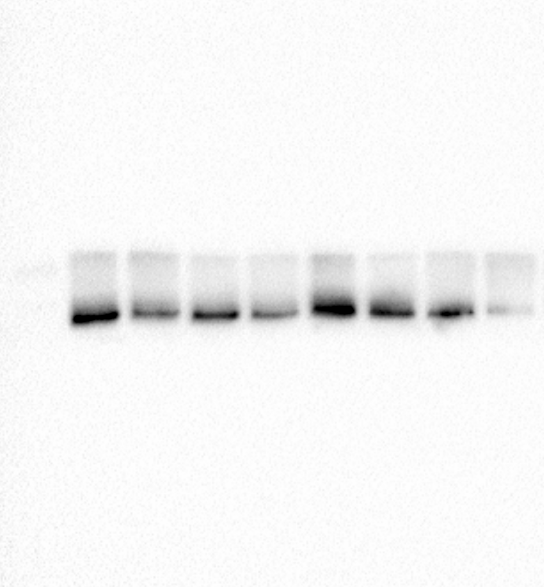


Figure 1C ACTB-2 Figure 1C NSUN2-2


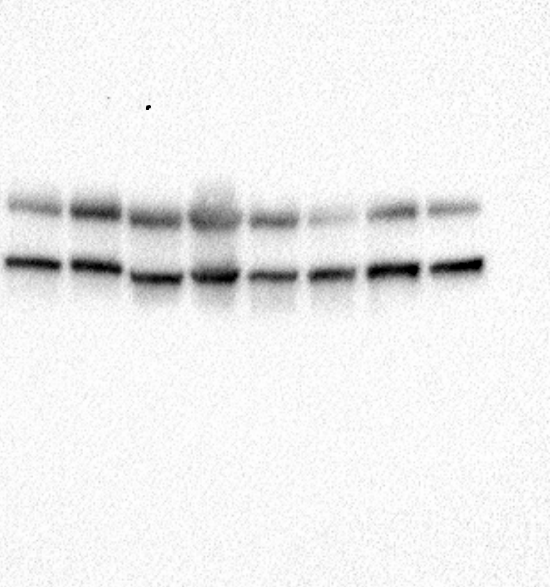

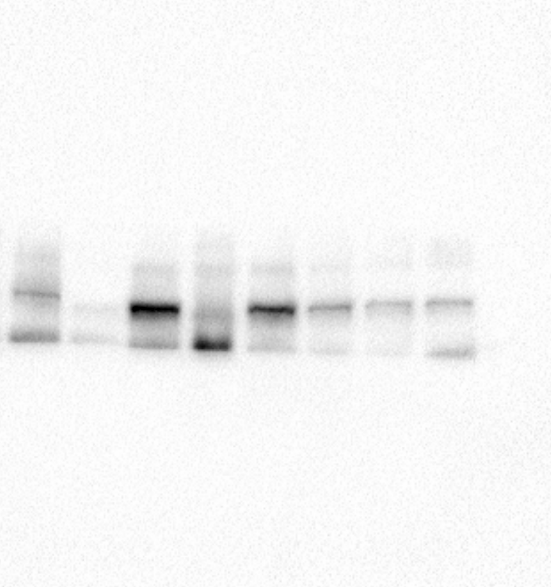


Figure 1C ACTB-3 Figure 1C NSUN2-3


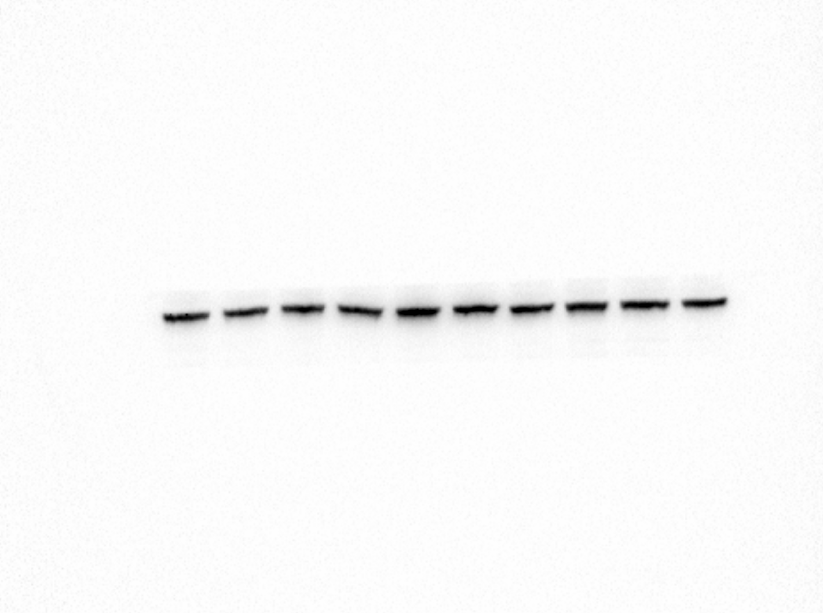


Figure 4E ACTB


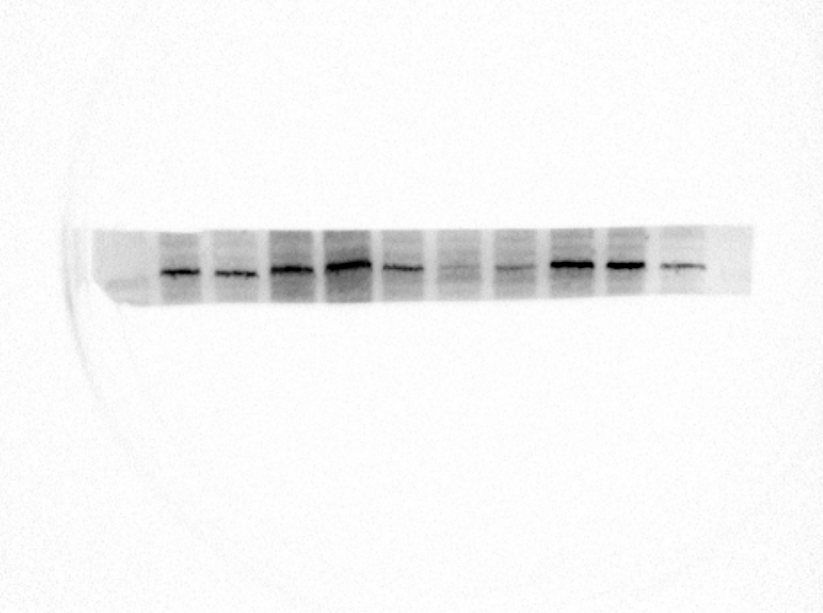


Figure 4E PKM2


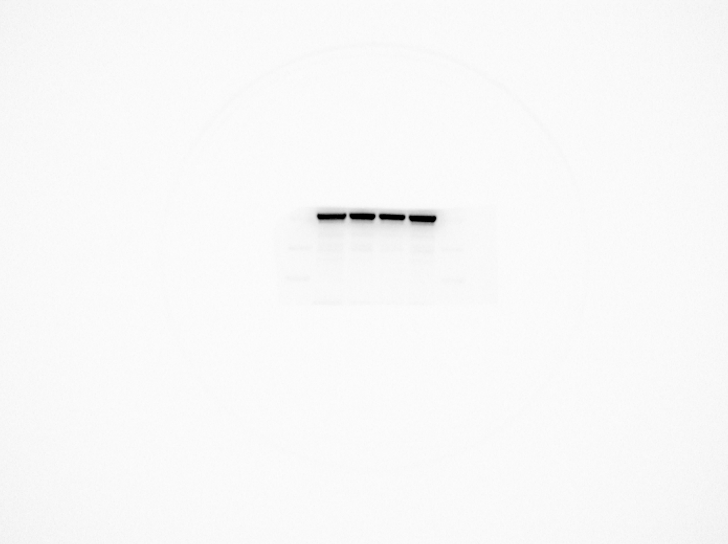


Figure 6E ACTB


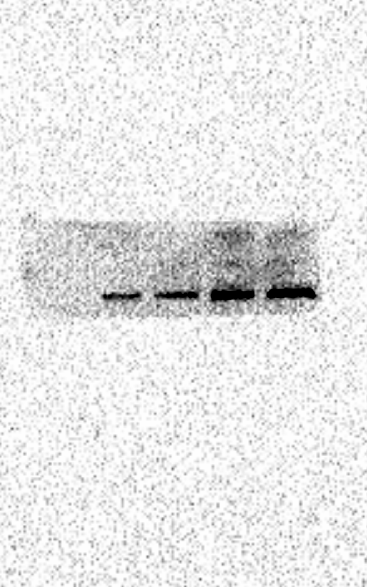


Figure 6E NSUN2

Marker


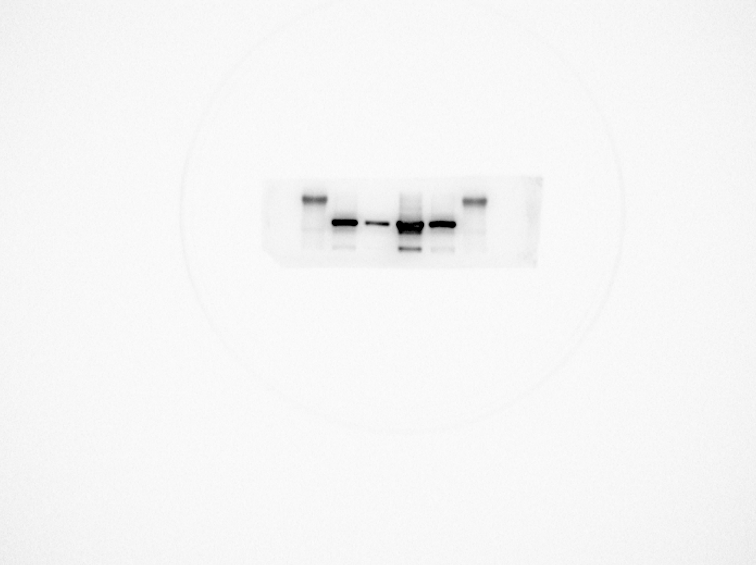


Figure 6E PKM2 Monomer

Marker


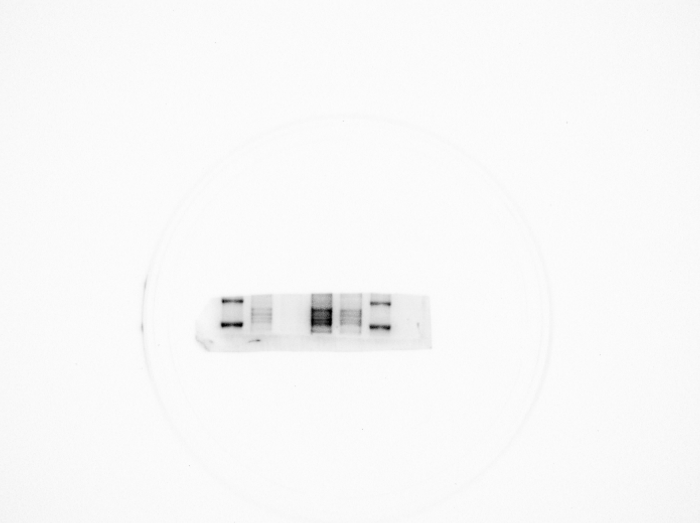


Figure 6E PKM2 Dimer

Marker


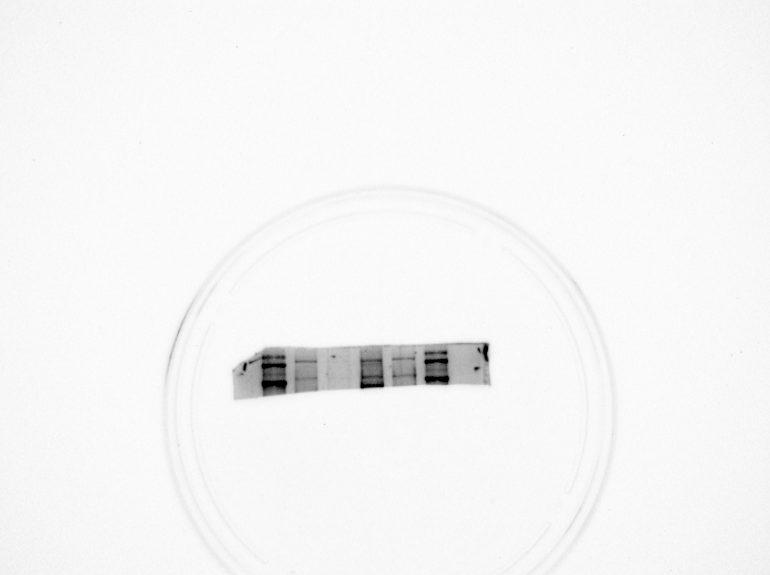


Figure 6E PKM2 Tetramer


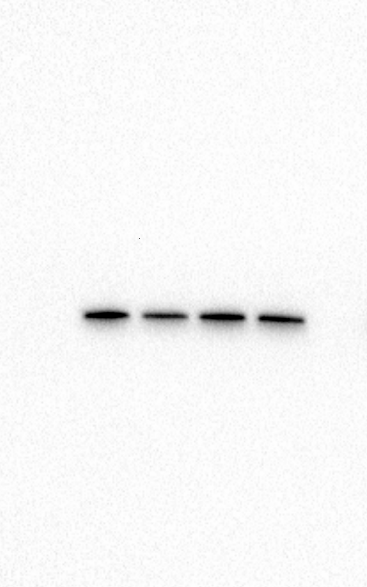


Supplemental Figure 2C Hep3B-ACTB


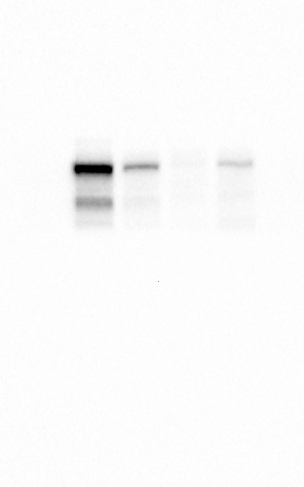


Supplemental Figure 2C Hep3B-NSUN2


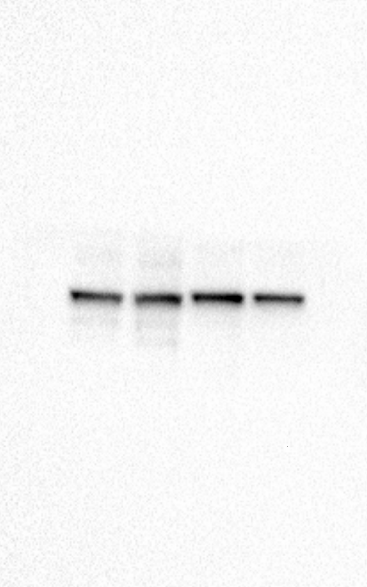


Supplemental Figure 2C HepG2 SNU387-ACTB


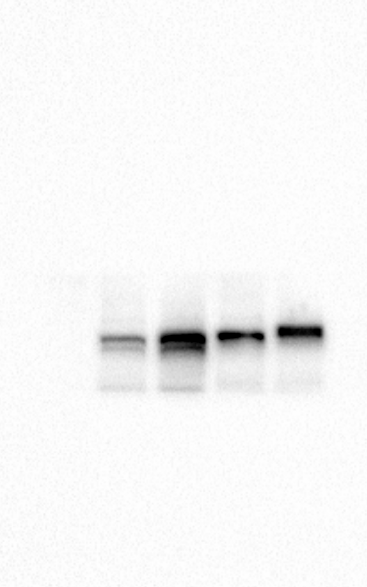


Supplemental Figure 2C HepG2 SNU387-NSUN2


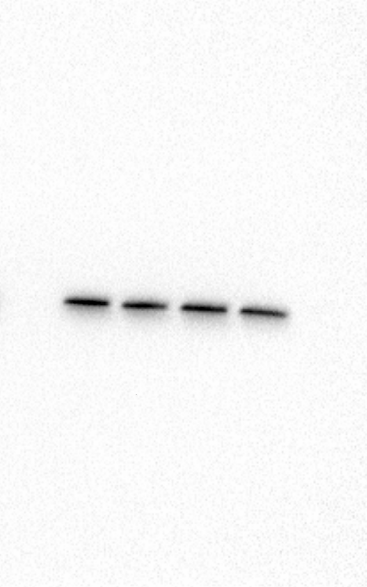


Supplemental Figure 2C Huh7-ACTB


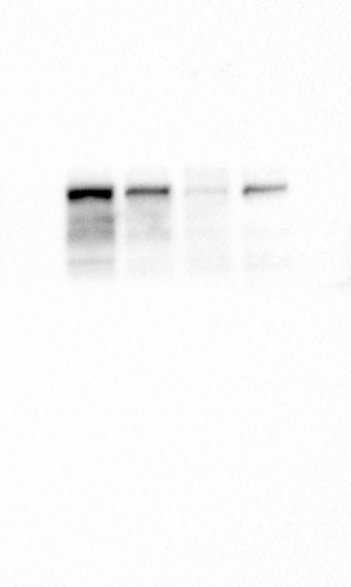


Supplemental Figure 2C Huh7-NSUN2
