# Supplementary material for: The RNA M5C methyltransferase NSUN2 promotes progression of hepatocellular carcinoma by enhancing PKM2-mediated glycolysis
Source: Cell Death Dis. 2025 Feb 9;16(1):82. doi: 10.1038/s41419-025-07414-5 (PMC11808121; doi:10.1038/s41419-025-07414-5)
Supplement: Supplementary file 2 — Supplementary Figures 1 and 2 and Supplementary Tables 1–3 [file 41419_2025_7414_MOESM2_ESM.doc]

**The RNA m5C methyltransferase NSUN2 promotes progression of hepatocellular carcinoma by enhancing PKM2-mediated glycolysis**

Qin Qi^1#^, Rui Zhong^1#^, Yan Huang^1#^, Yong Tang^2#^, Xiao-wen Zhang^1^, Chang Liu^3^, Chun-fang Gao^1, 4^, Lin Zhou^3*^, Jian Yu^5*^, Lu-yi Wu^1*^

^1^Yueyang Hospital of Integrated Traditional Chinese and Western Medicine, Shanghai University of Traditional Chinese Medicine, Shanghai 200437, China

^2^International Joint Research Centre on Purinergic Signalling, Chengdu University of Traditional Chinese Medicine, Chengdu 610075, China

^3^Department of Laboratory Medicine, Changzheng Hospital, Naval Medical University, Shanghai 200003, China

^4^Department of Laboratory Medicine, Eastern Hepatobiliary Surgery Hospital, Naval Medical University, Shanghai 200438, China

^5^The Third Department of Hepatic Surgery, Eastern Hepatobiliary Surgery Hospital, Naval Medical University, Shanghai 200438, China

^*^Corresponding authors.

^#^Contributed equally.

Correspondence to Professor Luyi Wu, Yueyang Hospital of Integrated Traditional Chinese and Western Medicine, Shanghai University of Traditional Chinese Medicine, 110 Ganhe Road, Shanghai 200437, China, [wuluyi@shutcm.edu.cn](mailto:wuluyi@shutcm.edu.cn); Doctor Jian Yu, The Third Department of Hepatic Surgery, Eastern Hepatobiliary Surgery Hospital, Naval Medical University, 225 Changhai Road, Shanghai 200438, China, [1274432278@qq.com; and](mailto:1274432278@qq.com; or) Professor Lin Zhou, Department of Laboratory Medicine, Changzheng Hospital, Naval Medical University, 415 Fengyang Road, Shanghai 200003, China, [lynnzhou36@126.com](mailto:lynnzhou36@126.com).

(Supplementary Tables. 4-12 are presented in Supplementary Data 2)

**Table of contents**

**Supplementary Fig. 1……………………………………………………………………2**

**Supplementary Fig. 2……………………………………………………………………3**

**Supplementary Table. 1 ...………………………………………………………………2**

**Supplementary Table. 2 ...………………………………………………………………5**

**Supplementary Table. 3 ...………………………………………………………………6**


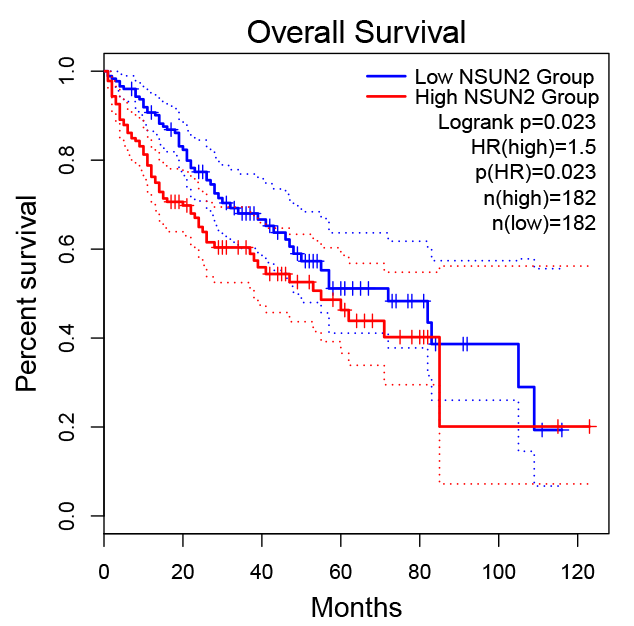


**Supplementary Fig 1. According to TCGA database in GEPIA2 (http://gepia2.cancer-pku.cn/#index), higher mRNA level of NSUN2 predicted poorer overall survival of HCC patients.**


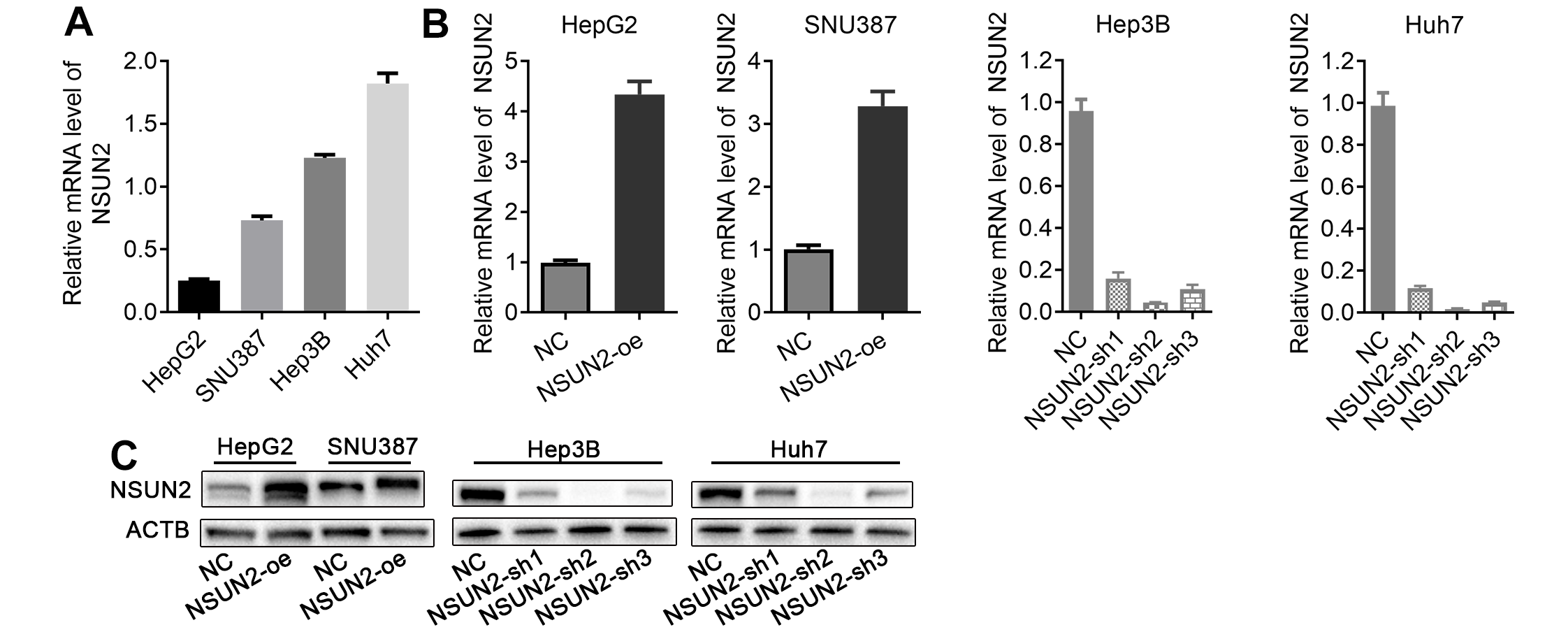


**Supplementary Fig 2. The establishment of HCC cells stably overexpressing or silencing NSUN2.**

(A) RT-qPCR showed the mRNA level of NSUN2 in different HCC cell lines. (B) RT-qPCR showed the mRNA level of NSUN2 in HCC cells after overexpressing or silencing NSUN2 using lentivirus. (C) Western blot showed the mRNA level of NSUN2 in HCC cells after overexpressing or silencing NSUN2 using lentivirus. oe, overpression; sh, short hairpin.

**Supplementary Table 1. Clinical Characteristics of 125 HCC Patients in this study.**

|  | HCC | | |
| --- | --- | --- | --- |
|  | Cohort 1 | Cohort 2 | Cohort 3 |
| All cases | 40 | 80 | 5 |
| Age, years,>50: ≤50 | 10:30 | 36:44 | 2:3 |
| Gender, male/female | 32:8 | 61:19 | 5:0 |
| HBsAg, positive/negative | 37:3 | 75:5 | 5:0 |
| Liver cirrhosis, with/without | 10:30 | 54:26 | 0:5 |
| AFP, µg/L,>20: ≤20 | 29:11 | 56:24 | 2:3 |
| Tumour size, cm,>5: ≤5 | 15:25 | 38:42 | 1:4 |
| No. tumour, multiple: solitary | 4:36 | 14:66 | 0:5 |
| Edmondson’s grade, III+IV: I+II | 34:6 | 67:13 | 1:4 |
| Microvascular invasion, present: absent | 14:26 | 45:35 | 2:3 |
| Pathological satellite, present/absent | 15:25 | 33:47 | 1:4 |
| Encapsulation, incomplete/complete | 31:9 | 52:28 | 4:1 |
| TNM stage, II+III: I | 14:26 | 16:64 | 0:5 |
| BCLC stage, B+C: A | 3:37 | 13:67 | 0:5 |

**Supplementary Table 2. The antibodies used in this study.**

| **Antibody** | **Supplier** | **Catalogue number** | **Host** | **Mono-/polyclonal** |
| --- | --- | --- | --- | --- |
| NSUN2 | Proteintech | 20854-1-AP | rabbit | poly |
| 5-methylcytosine (m5C) | Abcam | ab10805 | mouse | mono |
| PKM2 | Proteintech | 15822-1-AP | rabbit | poly |
| ACTB | Proteintech | 20536-1-AP | rabbit | poly |

**Supplementary Table 3. Primers and RNA sequences used in this study.**

| **primers for RT-qPCR** | |  |
| --- | --- | --- |
| Primer Name | Sequence (5'-3') | product size (bp) |
| ACTB-F | CCACCATGTACCCTGGCATTG | 289 |
| ACTB-R | TCATCTTGTTTTCTGCGCAAGTTA |
| NSUN2-F | GAACTTGCCTGGCACACAAAT | 127 |
| NSUN2-R | TGCTAACAGCTTCTTGACGACTA |
| NSUN6-F | TCTCAGCCCTTCATTTGACAGT | 189 |
| NSUN6-R | TCCAGTGCTATAACTTCTCCCTG |
| PKM2-F | GGAGAAACAGCCAAAGGGGA | 209 |
| PKM2-R | GTGAGGACGATTATGGCCCC |
| PKM2-m5CRIPqPCR-F | TAGGTTGGGACACCAGGGAA | 271 |
| PKM2-m5CRIPqPCR-R | CTGGCTGTTTCTTGACCCCA |
| PKM2-bisulfitePCR-F | GGTTGGGATATTAGGGAAGAAGAT | 155 |
| PKM2-bisulfitePCR-R | ACTCCAAAAACCTCCAATCCA |
| B3GNT3-F | TCCTCCTCTTCAGTCTGCTAGT | 150 |
| B3GNT3-R | CCGGGTGGGTGACCATAGA |
| CD7-F | AGGAACAGTCCCAAGGATGG | 237 |
| CD7-R | GCCACGAGCACAGTTTCTTTATC |
| EML2-F | GTGGCGGGAACCACTAAGG | 198 |
| EML2-R | CCACACCGAGAGCATGTGA |
| FOXC1-F | TGTTCGAGTCACAGAGGATCG | 122 |
| FOXC1-R | ACAGTCGTAGACGAAAGCTCC |
| GDF15-F | ACCTGCACCTGCGTATCTCT | 228 |
| GDF15-R | CGGACGAAGATTCTGCCAG |
| LRP4-F | GTGAGGAGGACGAGTTTCCCT | 181 |
| LRP4-R | TCACCGTCGCAGTACCAATG |
| MAPT-F | CCAAGTGTGGCTCATTAGGCA | 106 |
| MAPT-R | CCAATCTTCGACTGGACTCTGT |
| MCTP1-F | CCTTACCCCTAAAGAAGGAGAGT | 233 |
| MCTP1-R | GGCGTAAACTTTGGGTCTGAAA |
| PODXL-F | TCCCAGAATGCAACCCAGAC | 179 |
| PODXL-R | GGTGAGTCACTGGATACACCAA |
| SLC1A7-F | CCTCACCGTGGCGTACTAC | 214 |
| SLC1A7-R | GGTGCGGTACTGTTTGAATGT |
| **siRNA** |  |  |
| Si-PKM2 | UGCCAUCUACCACUUGCAATT |
| **shRNA** |  |  |
| NSUN2-sh1 | ccCAAGAATGAACGGCTTCAT |  |
| NSUN2-sh2 | gaGCGATGCCTTAGGATATTA |  |
| NSUN2-sh3 | caGTGGAAGGTAATGACGAAA |  |
